# Canine Leishmaniasis: Update on Epidemiology, Diagnosis, Treatment, and Prevention

**DOI:** 10.3390/vetsci9080387

**Published:** 2022-07-27

**Authors:** Manuel Morales-Yuste, Joaquina Martín-Sánchez, Victoriano Corpas-Lopez

**Affiliations:** Department of Parasitology, University of Granada, 18011 Granada, Spain; yuste@ugr.es (M.M.-Y.); joaquina@ugr.es (J.M.-S.)

**Keywords:** canine leishmaniasis, leishmaniasis, dog, *Leishmania infantum*, treatment, diagnosis, epidemiology

## Abstract

**Simple Summary:**

Dogs are the main host of *Leishmania infantum*, a parasite that causes an incurable disease called canine leishmaniasis. This parasite is transmitted through the bite of a sandfly (a small insect related to mosquitoes and flies) in tropical and subtropical countries, but direct transmission between dogs, and from pregnant dogs to their puppies, exists. We reviewed the advances in tools and techniques for the surveillance of the disease, its diagnosis, treatment, and prevention. Canine leishmaniasis is expanding to the Northern Hemisphere, where it is barely known, due to climate change and the importation of dogs. Surveillance is therefore necessary in order to determine the extent of the disease in these areas and to monitor the appearance of the sandflies. Molecular techniques and rapid serological tests are now widespread for diagnosis and epidemiological studies. Several vaccines have been developed in the last decade, and even though their efficacy is limited, these advances will pave the way for the development of better vaccines against *Leishmania* and other parasites. Although new pharmacological tools are available, we are still waiting for the ideal drug that can eliminate the parasite from target organs and limit transmission to sandflies, without the side effects of current antileishmanials.

**Abstract:**

Dog are the main reservoir of *Leishmania infantum*, causing canine leishmaniasis, an incurable multisystemic disease that leads to death in symptomatic dogs, when not treated. This parasite causes visceral, cutaneous, and mucosal leishmaniasis in people in the Mediterranean Basin, North Africa, South America, and West Asia. This disease is mostly unknown by veterinarians outside the endemic areas, but the disease is expanding in the Northern Hemisphere due to travel and climate change. New methodologies to study the epidemiology of the disease have found new hosts of leishmaniasis and drawn a completely new picture of the parasite biological cycle. Canine leishmaniasis diagnosis has evolved over the years through the analysis of new samples using novel molecular techniques. Given the neglected nature of leishmaniasis, progress in drug discovery is slow, and the few drugs that reach clinical stages in humans are unlikely to be commercialised for dogs, but several approaches have been developed to support chemotherapy. New-generation vaccines developed during the last decade are now widely used, along with novel prevention strategies. The implications of the epidemiology, diagnosis, treatment, and prevention of canine leishmaniasis are fundamental to public health.

## 1. Introduction

*Leishmania* constitutes a genus of widespread parasitic species that infect a variety of hosts through the bite of the sand fly, leading to a disease called leishmaniasis. In humans, *Leishmania* spp. causes mucosal, cutaneous, and visceral leishmaniasis. Dogs can be infected by a range of *Leishmania* species that cause visceral, cutaneous, or mucosal leishmaniasis as well [1,2], but the focus of this review is the disease caused by *Leishmania infantum*.

Canine leishmaniasis (CanL) is a multisystemic disease that affects dogs, the main reservoir of *L. infantum* [3]. This parasite is endemic in the Mediterranean basin, South America, and Central and Southwest Asia, being the most widespread *Leishmania* species.

## 2. Life Cycle, Hosts, and Vectors

The life cycle of *L. infantum* involves mammal hosts and sand fly hosts that act as vectors. Sand flies (Family *Phlebotomidae*) are small Diptera with limited flying ability that feed on plant juices. Sand flies are nocturnal insects and they are only active from dusk until the first hours of the night and at dawn. In temperate climates, sand flies are active from April to October in the Northern Hemisphere, whereas in tropical countries, they can be found throughout the whole year. Females also need blood in order to mature their eggs; thus, they take blood meals from vertebrates.

*L. infantum* vectors belong to the genera *Phlebotomus* (Europe, Africa, and Asia) and *Lutzomyia* (American continent). Eight *Phlebotomus* species have been implicated as *L. infantum* vectors in the Mediterranean subregion that includes southern Europe, North Africa, and parts of Asia: *Phlebotomus. perniciosus, P. ariasi, P. neglectus, P. kandelakii, P. perfiliewi, P. langeroni, P. tobbi*, and *P. balcanicus* [4]. On the other side of the Atlantic Ocean, *Lutzomyia Longipalpis, L. cruzi, L. evansi, L. migonei, L. forattinii, L. almerioi, L. whitmani*, and *L. fischeri* are associated with the transmission of *L. infantum*, and CanL has been reported in Argentina, Bolivia, Brazil, Colombia, French Guyana, Mexico, Paraguay, Uruguay, and Venezuela.

When female sand flies take a blood meal, they ingest infected macrophages containing intracellular amastigotes (macrophages are the host cell in mammals) that transform into promastigotes in the insect digestive tube. Unlike amastigotes, promastigotes have a motile flagellum that help them migrate to the sand fly stomodeal valve when they reach the metacyclic promastigote stage. These metacyclic promastigotes are highly infective to mammals when the sand fly takes further blood meals.

Although dogs are the main reservoir hosts of *L. infantum*, the parasite has been found in cats [5], rabbits, hares, and wild rodents [6,7]. Other wild animals have also been reported as *L. infantum* hosts: wolves (*Canis lupus*), foxes (*Vulpes vulpes*), jackals (*Canis aureus*), and genets (*Genetta genetta*) [8].

## 3. The Disease in Dogs

### 3.1. Disease Mechanisms and Immune Response

Upon a blood meal, dog macrophages phagocytises inoculated *L. infantum* metacyclic promastigotes, and they start their transformation into amastigotes that multiply. Parasites are able to avoid the dog innate immune response through the remodelling of the parasitophorous vacuole, hindering the macrophage signalling pathways to their advantage [9]. Once the parasitic burden is too large, macrophages burst, releasing amastigotes that are phagocytised by other cells of the mononuclear phagocyte system, mainly in the blood, liver, spleen, and bone marrow.

A mixed immune response is usually generated in Leishmania infections, both in human and canine leishmaniasis, which results in the accumulation of Th1 (cellular response) and Th2 (humoral response) cytokines in a delicate balance that once broken, will lead to disease elimination or progression [10]. Th1 cytokines, such as interferon gamma (IFN-γ), tumor necrosis factor alpha (TNF-α), and interleukin 2 (IL2), activate the macrophage that eliminates the parasite through the generation of nitric oxide (NO) and other oxidative species. On the other hand, IL10, IL13, IL4, and transforming growth factor beta (TGF-β) lead to a humoral response, in which immunoglobulins are vastly synthesized, the macrophage is inactivated, and the parasite can thrive. According to some authors, this Th1/Th2 dichotomy can be seen in immunoglobulin subtypes IgG1 and IgG2, associated with Th2 and Th1 response, respectively, and reported IgG1 prevalence in symptomatic dogs [11,12]. Other authors have not observed this dichotomy [13]. Recently, there has been a great interest in the involvement of toll-like receptors (TLRs) in the immunopathogenesis of CanL. TLRs are one of the most important pattern recognition receptor molecules that recognize microbial pathogens and induce an inflammatory response. In a recent study, downregulation of TLR7 and IFN-γ was found in unaffected skin in infected dogs when compared with skin from more severely affected dogs [14]. TLR2 seem to be associated with disease progression and has been found significantly upregulated in damaged skin of sick dogs when compared with healthy skin of non-infected dogs, as well as documented in other tissues, such as the intestine, brain, peripheral lymphoid organs, liver, blood, and skin [14].

### 3.2. Clinical Signs

*L. infantum*-infected macrophages can be found in parasitized tissues, causing granulomatous inflammatory reactions that lead to most symptomatology. Most dogs present with poor body condition, or cachexia; they are usually thin and tend to anorexia. Main skin signs include skin peeling, cutaneous lesions (nodular, ulcerative, and pustular), and exfoliative dermatitis. Alopecia, pale mucosa, and erythematous reactions are also common. Onychogryphosis, or nail enlargement, is very common and is associated with lichenoid and interface mononuclear dermatitis in the absence of parasites [9]. Ocular damage can also be found, as blepharitis, uveitis, and conjunctivitis are very common. Adenopathy is common in symptomatic dogs and is characterized by lymph node enlargement as a consequence of hypertrophy in the node structure. This enlargement is not usually correlated with parasite load in the tissue or the clinical state of the animal [9,15].

Kidney involvement appears in most infected dogs: glomerulonephritis is associated with immune complex deposition and can progress to kidney failure. Kidney damage can start at the beginning of the infection, but only manifests in advance stages through proteinuria and high blood creatinine levels. Renal failure is the main death cause in CanL [16].

CanL dogs usually present with epistaxis, haematuria and haemorrhagic diarrhoea due to the coagulation disorders of the disease [9].

The presence of amastigotes in the spleen leads to spleen enlargement or splenomegaly due to the macrophage infiltration and changes in the microstructure of this organ. A similar pattern can be found in the liver, and some cases progress to hepatitis [17]. In CanL, the spleen is one of the most affected organs during infection, along with the skin and bone marrow. The spleen concentrates high parasite burden and, as a consequence, presents significant morphological changes, including hypertrophy and hyperplasia of the red pulp and infiltration of mononuclear and plasma cells. The white pulp of the spleen presents macrophage replacement by lymphocytes due to hypertrophy and hyperplasia of this region [18,19]. The spleen is the organ where the response to the parasite is established and where the process of cell activation occurs [20,21]. Involvement of innate immune sensors in the immune response against *L. infantum* has been found: TLR-5 and TLR-9 play a role in splenic innate immunity during CanL [22].

The pancreas can be also affected in CanL, but the detection rate and parasite burden are usually low [23]. A study demonstrated that *L. infantum* is one of the aetiological agents of chronic pancreatitis in dogs and found association of poor body condition and cachexia with concomitant infection of the pancreas, spleen, and/or bone marrow, and suggested that these manifestations are the result of a more advanced stage of canine visceral leishmaniasis [23].

*L. infantum* invades most dogs’ tissues and organs [24], reaching the bone marrow (a densely parasitized organ), which is considered the deepest organ and the one responsible for the persistence of the disease, including relapses. Although haematopoiesis is normal in early disease stages, bone marrow parasitization brings about changes in cell production, leading to pancytopenia and non-regenerative anaemia, histiocytic hyperplasia, and erythrocytic hypoplasia, ending in medullar aplasia. All these changes translate into haematological and coagulation disorders that are worsened by haemolysis alterations in association with spleen damage [25].

Skin is a key organ in CanL, since the presence of parasites here is necessary for the transmission to the vector. Skin parasite load, although variable, seemed to increase with disease severity, and it is also well correlated with the vector infectivity [26]. This variability seemed to be associated with the hypothesis that some dogs are super-spreaders and present very high skin parasite loads. The importance of skin parasite load is highlighted by the fact that even in vertical transmission, dogs present high skin parasite burden and infectiousness to the vector [27].

The liver is one of the target organs in CanL, where *L. infantum* establishes itself through the invasion of the resident macrophages, or Kupffer cells. Parasite infection leads to inflammatory changes in the liver, including the formation and maturation of granulomas in this organ that could be associated with the resolution of the infection. The liver also has some immune functions, such as the removal of pathogens and their antigens from the circulation, and hepatocytes have been found to coordinate this function during CanL through the activation of inflammatory mechanisms [28,29,30].

## 4. Epidemiology

Most epidemiological studies on CanL have been carried out using serological techniques, due to their ease of use and efficiency. However, it is widely acknowledged that these immunological methods are limited by their inability to distinguish between past and present infections, as well as by the possibility of cross-reactions with other infectious agents. Significant cross-reactions have been reported in individuals infected *with Trypanosoma cruzi* [31]. IFAT is one of the most frequently used immunological methods, using 1/80 or 1/160 titre thresholds. When quantitative serological methods are used in epidemiological surveys, a significant number of animals display titres below the positivity threshold, known as uncertain titres, which could be due to periods of pre-patent infection, remission stages, the appearance of non-specific cross-reactions, and to latent forms of the disease. Uncertain titres are more frequent in asymptomatic than in symptomatic dogs. The proportion of asymptomatic dogs is high in endemic regions, favouring the inadvertent spread of *L. infantum* in the dog population and making the early detection of these asymptomatic carriers crucial for the reduction in prevalence figures [32,33]. In the Alpes-Maritimes region (France), 50% of seropositive animals showed clinical signs of infection at the time of testing [34].

Prevalence values from epidemiological studies in which dogs are randomly sampled are considerably influenced by the diagnostic technique used. The highest prevalence values are frequently produced by PCR techniques that show low correlations with serological methods. Correlation found between serological methods, such as Q letitest, ELISA, and Kalazar Detect^TM^, is also low [33]. On the contrary, high concordance has been found between ELISA using a K9-K39-K26 recombinant chimeric antigen and IFAT [31].

IFAT is frequently used as a reference test to estimate the true prevalence of CanL in the Mediterranean Basin. Other groups prefer an in-house ELISA technique, claiming that it is easier to perform and interpret. Both serological techniques are recommended by the World Organization for Animal Health for CanL surveillance studies and to determine prevalence of infection [31].

The Mediterranean basin is an endemic region for CanL, where it represents a major veterinary problem and raises human health concerns. However, the distribution of the disease is heterogeneous, and not all countries and locations have been equally studied and characterized.

In southern European countries, CanL seroprevalence reaches high levels. Early detection of infected animals may be critical in controlling the spread of the disease. CanL is widespread in Spain, and it can be considered endemic in almost the entire territory. In the southeast section of the country, the seroprevalence was calculated at 23.7%, using both blood samples from veterinary clinics [35] and randomly selected dogs [33], and hot spots have been found in the Axarquia region (Malaga) [36]. Figures are lower in central Spain (1–5%) [37] and in the North [38,39], whereas in the Pyrenees and northeastern areas, 19.5% prevalence was found, mainly among the hunting dog population [40]. Low prevalence numbers (0–2.5%) were found in the Canary Islands, although imported cases were not ruled out [38,39,41].

In other Southern European countries, epidemiological surveys show similar varying patterns. In Italy, a 14% prevalence was found in the highly endemic regions of the Campania region, which is considered highly endemic, whereas in France, a 12% average prevalence was found, where values ranged from 8.1 to 28% in the areas closest to the Mediterranean Sea. In southeastern of France, the prevalence of symptomatic CanL in military dogs was very low in 2009 (0.7%), whereas in 1993, it was 3.5%, probably due to the recent use of deltamethrin-impregnated collars. An overall prevalence of 6.31% has been found in Portugal, ranging from 0.88 to 16.16%, with the highest prevalence in the interior regions [34,42,43,44].

It has been suggested that the distribution area of sand flies may expand, both in terms of latitude and altitude, as a consequence of global warming, bringing about an increase in leishmaniasis prevalence. The geographical expansion of *L. infantum* vectors has been reported in northern Italy [45], or The Pyrenees in Spain [46], and this has been attributed to climate change. In Sierra Nevada (southern Spain), Barón et al. (2011) demonstrated the presence of sand flies at higher altitudes than previously reported [47], and Martín-Sánchez et al. (2009) detected an increase in the seroprevalence of canine leishmaniasis over a period of 22 years, which may be associated with climate change [48]. In Italy, the spread of CanL and its vectors to the north has been monitored. New CanL foci and the presence of competent vectors were reported in the northern regions of the country, where autochthonous cases had not been reported previously, and seroprevalence in resident dogs was 4–6% [49]. Global warming is a possible cause of the spread of the disease to cooler areas, and the increased movement of infected animals from areas where the disease has traditionally been endemic may facilitate this process, along with the spread of sand fly vectors. In Portugal, CanL seroprevalence has increased from 4–7% in the 1990s, to a prevalence rate of up to 20% found in endemic foci [50]. In southern Spain, *L. infantum* transmission at high altitudes (1753–1813 m a.s.l.) was confirmed by high CanL prevalence (23%), which entails an increase in the leishmaniasis risk area, driven by sand fly colonization [51].

CanL is endemic in Greece, where both *L. infantum* and *L. tropica* (causative agents of cutaneous leishmaniasis) are present [52]. In a study in 2005–2010, average dog seropositivity was 22.1% (reaching >50% in some areas), and infected animals were found in 43 of 54 prefectures [52]. In a more recent study among asymptomatic dogs, using a different serological method (Speed Leish K), average seropositivity among the dog population was 13.8%, and infected dogs were found in all prefectures, reaching >50% in some areas in the north of the country [53].

Canine leishmaniasis in the Balkans was recently reviewed by Vaselek (2021) [54], where a number of reports during the past decade indicate the emergence of autochthonous canine leishmaniasis across this region. A total of 3.2% seroprevalence was found in Albania in the latest report, and several studies have also been carried out in Bosnia-Herzegovina (16.7% seroprevalence, 3.1% PCR) and Kosovo (18% seroprevalence). CanL has also been found in Croatia (1.38% seroprevalence among 400 dogs), Montenegro (with >5% seroprevalence in the latest survey, although most studies have been carried out in symptomatic dogs), North Macedonia, and Serbia, where seroprevalences between 1.8–10.6% have been reported. Data is very scarce in Slovenia, a country bordering endemic countries, but the first autochthonous case was reported in 2014, and a recent survey found a 1.8% seroprevalence [55].

Although the disease is not endemic in Central Europe, *P. mascittii* is present in Germany and has expanded through Austria in the past decade [56,57]. The vector competence of this species is still unclear [57]. *P. perniciosus* has been found in southwest Germany, but vector-borne transmission has not been detected to date in the country; however, vertical, direct, and venereal transmission from imported dogs have been reported in Germany and other Central European countries, such as the Czech Republic, where *P. mascittii* is endemic [58,59,60], in Western European countries, such as Belgium and the Netherlands, and in the United Kingdom, where sand flies have not been reported [61]. As reviewed by Mihalca et al. in 2019, CanL was not considered endemic in most Eastern European countries, with only sporadic cases reported. However, new imported or autochthonous cases are now reported every year. CanL cases have been found in Romania, where 3.7% seropositivity has been recently reported (8.7% by PCR) [62]; in Hungary, where the only CanL case was reported in 2012; and in Bulgaria, where 10 cases have been reported in the last decade, but the seroprevalence studies carried out in these countries were unclear [62]. Few or no epidemiological studies have been carried out in Slovakia, Poland, Belarus, Moldova, and Ukraine [62].

CanL was found in the United States in the 1980s, and several autochthonous vertical transmission events have been reported since then [63]; however, the main vector (*L. longipalpis*) has not been reported there, but other sandflies, such as *L. shannoni*, may be able to transmit the parasites [64].

## 5. Diagnosis

Parasitological diagnosis is always a laboratory diagnosis. Clinical signs and/or clinicopathological abnormalities compatible with disease leads us to suspect that the animal has canine leishmaniasis (CanL). Confirmation of the aetiology of the infection can be obtained using different direct and indirect laboratory diagnostic methods: parasitological, molecular, and serological.

Parasitological methods include microscopic examination and culture. Microscopic observation of amastigotes in stained smears of the bone marrow or lymph node is a conclusive diagnosis, but this requires experience and time. Sensitivities of 52–85% for bone marrow, and 52–58% for lymph node aspirates, have been found [31]. Reports describing the presence of amastigotes in nodular masses with atypical localization, such as tongue, testis, and oral or nasal masses, have been reported after fine-needle aspiration [65]. Histopathological (sections stained with haematoxylin-eosin) and immunohistochemical approaches in formalin-fixed paraffin-embedded (FFPE) tissues requires considerable expertise and training, and these do not provide an increase in sensitivity. These methods can also yield false positive results because artefacts can be erroneously considered as amastigotes [66]. Compared with cytology, histology is more laborious and time-consuming, and the identification of amastigotes may be more difficult. On the other hand, histology has the advantage of providing additional information on the cytoarchitectural pattern of the lesions, allowing the discrimination between dogs in which the parasite is associated with typical lesions and those in which the infection does not seem to be associated with the disease [65]. If the results are negative, the biopsy sample can be used for molecular analyses [31].

In vitro culture is not a simple procedure, and it requires at least a month to provide a negative result; therefore, it is currently not usually used as a diagnostic method. Its frequent use at the end of the last century was related to the need to use isoenzyme electrophoresis to identify *Leishmania* strains [67,68,69,70]. In addition, there is extensive variability in the growth rate among parasite strains and in parasite load among tissues. The popliteal lymph node is the most accessible biological material for culture, providing 64–100% positive results in symptomatic dogs and dogs with antibody titres ≥80 [32,67,71]. For isolation, it is recommended to use biphasic blood-agar media, such as NNN (Novy-McNeal-Nicolle) or EMTM (Evans’ modified Tobie’s medium), which can be supplemented with RPMI-1640 as a liquid phase instead of using liquid media [15,67].

Although parasite isolation in laboratory animals and xenodiagnoses have been used to solve research or epidemiological questions, these are rarely used in routine diagnosis [65,66].

Molecular diagnosis represents an improvement in sensitivity. Polymerase chain reaction (PCR) currently constitutes a sensitive tool for the diagnosis of CanL and the identification of *Leishmania* species; other methods, such as LAMP (loop-mediated isothermal amplification), are not commonly used. Many PCR assays have been described, but the selection of the most suitable PCR technique is complex. Sensitivity and specificity vary according to the PCR variant (conventional PCR, nested PCR, PCR-ELISA, and real time PCR) and the target DNA sequence (highly repeated sequences, such as kinetoplast DNA minicircles or small subunit ribosomal RNA genes, as well as a variety of unique genes).

One of the advantages of PCR is the variety of samples that can be analysed, including FFPE biopsies, blood, conjunctival, and oral swabs, and hair [15,72,73,74] and other non-invasive samples. The highest mean parasite load is found in the bone marrow, followed by the lymph node [15]. Parasitaemia is usually low, even in dogs with severe leishmaniasis [75].

Table 1 shows the sensitivity and specificity values of common invasive and non-invasive tissues using PCR techniques.

Compared with conventional PCR or nested PCR, rtPCR offers several advantages: it is less time consuming, and it can be converted into quantitative PCR. On the other hand, it is not necessarily more sensitive. rtPCR is carried out in closed systems that allow the amplification to be visualised in real-time, while being less prone to contamination. For qPCR, the parasite load can be obtained by interpolating the threshold cycle values obtained for each biological sample in a previously built calibration curve.

Another important issue is related to the specificity of the technique that is related to the DNA target; some PCRs are specific for the genus *Leishmania*, and only a few are specific for *L. infantum*. PCR primers targeting the minicircle kinetoplast DNA (kDNA) and internal transcribed spacer 1 (ITS-1) ribosomal DNA are amongst those most commonly used. The selection of the PCR to use will be influenced by the epidemiological scenario and the associated *Leishmania* species [74].

Nested PCR-RFLP for ITS-1 rDNA shows good diagnostic values and allows for the identification of almost all medically relevant *Leishmania* species with the HaeIII restriction enzyme, although MnII produces greater differences in molecular weights, and it should be recommended [74]. It was able to detect parasites in 29/46 (63%) conjunctival samples corresponding to 17/23 dogs (74%) [76]. Similarly, 25/36 dogs (69%) with lymphadenomegaly were diagnosed as positive by lymph node PCR, and 15/25 dogs (60%) with splenomegaly were determined as PCR-positive by spleen ITS-1 PCR, whereas blood sensitivity was limited (9%) [77].

Positivities determined by the kDNA-based methods are significantly higher. A real-time assay based on the amplification of a 120-base-pair conserved fragment of the kDNA minicircle was described by Francino et al. in 2006 [78]. The limit of detection was 0.001 parasites in the PCR reaction, and parasitaemia detection ranged from less than 1 to 107 parasites/mL, without differentiation between *Leishmania* species.

A PCR-ELISA based on the amplification of a fragment belonging to the variable region of the *L. infantum* kDNA minicircle is specific for detection of this species [79]. This PCR assay has a higher sensitivity than the other techniques used (IFAT, parasite cultures, and optical microscopy of stained samples) and permits detection of 1 fg of genomic DNA and a wide range of dog parasitaemia. There was 100% agreement between the results obtained with PCR-ELISA using DNA from blood samples and DNA from lymph node material, and 88% between those obtained from blood and bone-marrow samples from the same dogs [79]. Both kDNA PCRs have been widely used in diagnostic and epidemiological studies. A quantitative rtPCR related to this PCR-ELISA has been developed. Its sensitivity is slightly lower, but it has the advantages of real-time PCR and the ability to allow quantification [15,80]. A multiplex PCR format that allows differentiation between *L. infantum*, *L. tropica*, and *L. major* is also available [81]. The quantitative PCR is very useful for the diagnosis of CanL and facilitates the monitoring of the parasite load during and after treatment in different samples, allowing for the prediction of recurrences associated with residual parasites after treatment [80,82].

The specific humoral response in CanL is, in general, very intense, with high levels of specific immunoglobulins allowing for serological diagnosis; in addition, seroconversion occurs within a few months of infection [31]. However, the presence of anti-*Leishmania* antibodies alone is not a conclusive sign of disease due to the inability to discriminate between immunity and actual infectiousness. Therefore, to improve CanL diagnosis, it is advisable to combine IFAT with other non-immunological testing, to perform more than one serological test, or to continue monitoring with repeated immunological tests [33,66,83]. Several serological tests, with a variety of antigens and different cut-off titres, have been used for individual diagnosis, as well as for epidemiology surveys. Among these, IFAT uses whole body parasites as the antigen and is considered the “gold standard” of serologic diagnosis, being extensively used for the routine diagnosis of clinical cases in the veterinary field, as well as for epidemiological surveys.

In 2016, Adel et al. reviewed the diagnostic accuracy of IFAT in CanL, with reference to its sensitivity and specificity, through a meta-analysis. The sensitivity of IFAT was estimated at 90% and 31% in symptomatic and asymptomatic dogs, respectively. The specificity was estimated in non-endemic and endemic areas as 98% and 97%, respectively [84]. According to OIE, 2021, in a Chagas disease-free areas, IFAT has a sensitivity of 96% and a specificity of 98% [31].

Enzyme-linked immunosorbent assay (ELISA) is also widely used for laboratory diagnosis or field applications. The use of parasite-soluble extracts as the antigen limits their specificity [66]. Even so, an in-house ELISA technique, using sonicated whole promastigotes, has been frequently used by research groups for seroprevalence surveys [40]. ELISA can be carried out on serum or blood samples that are tested at a single dilution, which has been previously established to have an acceptable sensitivity (86 to 99%) and specificity [31]. A whole series of proteins specific to *Leishmania*, most of them conserved in evolutionary terms, have been characterised for more specific diagnostic methods. Two of these are the chimeric protein “Q,” which contains antigenic determinants of the antigens LiP2a, LiP2b, LiPO, and LiH2A, and the recombinant protein K39 (rK39), which contains a repeat sequence that is highly conserved among members of the *L. donovani* complex. Both recombinant antigens have been frequently evaluated as diagnostic markers for CanL [33,83,85]. rK39-based ELISA showed better diagnostic performance than rK28- and rKR95-based ELISA assays [86]. They are both recombinant kinesin-derived antigens from *L. infantum*, known like rK39 and rKDDR. The main difference between these last two antigens is the size of the non-repetitive kinesin region and the number of repetitions of the 39 amino acid degenerate motif (6.5 and 8.5, respectively). Recently, rKDDR-plus antigen, containing 15.3 repeats of the 39 amino acid degenerate motif, was evaluated by ELISA using dog sera; all three antigens showed a sensitivity of 98%, whereas the specificity of rKDDR-plus, fKDDR, and rK39 was 98%, 91%, and 83%, respectively [87]. These and other recombinant antigens can also be used as immunochromatographic rapid tests. The rapid immunochromatographic assay is easy to carry out and can be performed in veterinary clinics, but it has lower diagnostic sensitivity (30–70%) than IFAT and ELISA, and the specificity is medium-high; therefore, false negative results are common; this test does not allow for the evaluation of antibody titres [31].

Morales-Yuste et al., 2012, analysed 71 dogs with symptomatology compatible with CanL using 3 serological techniques (IFAT, ELISA with protein Q antigen —Q letitest ELISA—, and the rapid immunochromatographic test using rK39 antigen —Kalaazar Detect^TM^— and found a higher correlation between the two commercial techniques (69%). IFAT, with a threshold of 1/160, was the most sensitive diagnostic technique, showing 77.5% positivity and 22.5% uncertain titres. Using IFAT as a reference, sensitivity and specificity was 71% and 94%, respectively, for Kalaazar Detect^TM^, and 60% and 88%, respectively, for Q letitest ELISA [33].

## 6. Treatment

The aim of CanL treatment is to control clinical signs and alterations, improve the dog’s cell immunity, reduce the parasite burden, avoid relapses, and decrease the transmission rate to the vector [88]. Sterile cure is extremely difficult in CanL, but the parasite load can be reduced to asymptomatic levels using chemotherapy [89]. The antileishmanial arsenal is similar to visceral leishmaniasis in humans, with some exceptions. In Latin America, treatment of dogs with leishmaniasis is not usually performed, since dog culling is recommended after diagnosis in most countries. In Brazil, treatment of leishmaniotic dogs had been forbidden until 2017, when miltefosine was approved [90].

Therapy choices must be based on the disease stage; Solano-Gallego et al. (2011) established a staging system for dogs that classified the disease in four stages: stage I (asymptomatic to mild disease), stage II (moderate disease), stage III (severe disease associated with chronic kidney disease), and stage IV (very severe disease that included nephrotic syndrome). Stage I dogs can be left untreated or treated with allopurinol alone, whereas it is recommended to treat stage II and III dogs with combinations of allopurinol plus antimonials/miltefosine. Stage IV dogs should be treated with allopurinol alone, in order to avoid further kidney damage [91]. Therapy includes chronic kidney disease management [92].

The use of allopurinol is widespread, given its leishmaniostatic potential and low toxicity that make it very effective in dogs with kidney damage. Allopurinol is frequently combined with other drugs (particularly antimonials), but can be used as a monotherapy in asymptomatic dogs, or as a follow-up or even as a preventive treatment [93]. Allopurinol is a hypoxanthine analogue that blocks xanthine oxidase, disrupting purine metabolism. It is usually well tolerated, and its only side effect is urolithiasis [94]. Although allopurinol resistance had not been reported earlier, recent clinical and experimental evidence suggests that it is possible and could be associated to a decrease in S-adenosylmethionine synthetase copy numbers [95,96,97].

Antimonial therapy (meglumine antimoniate and sodium stibogluconate) is the first line treatment in Mediterranean countries, in spite of their high toxicity, and its combination with allopurinol is the therapy of choice in this region. These drugs have been used for more than 50 years in human visceral leishmaniasis, but they are being replaced by amphotericin B in the Mediterranean Basin. Pentavalent antimonials are prodrugs that are metabolised to trivalent antimonium, the active molecule whose mechanism of action is not completely known, but seems to be linked to DNA damage and fatty acid oxidation.

Resistance to antimonials in CanL has been reported in the past [98], and the appearance and spread of antimony-resistant strains is expected, given the wide use of meglumine antimoniate in dogs and a possible non-rational use of this drug in veterinary practice. In this study, treatment with meglumine antimoniate was utterly ineffective; this outcome might partially be due to resistance phenomena in these dogs with naturally acquired CanL, all of them from an endemic area of leishmaniasis due to *L. infantum*, where meglumine antimoniate is widely used and the spread of antimony-resistant strains is likely to take place, as reported previously [99,100]. Combination therapy can help recycle antimonial compounds to continue their use, limiting the appearance of resistance [101]. Another strategy is the use of nanoencapsulation in order to improve the ability of the drug to target the parasite and avoid undesirable effects in dogs [102,103,104].

Miltefosine was the first oral antileishmanial drug; initially developed as an anticancer drug, it was found effective in the treatment of human visceral leishmaniasis [105,106]. Its mechanism of action is associated to lipid binding and apoptosis triggering [106]. Miltefosine has been successfully used in monotherapy [107,108], and in combination with allopurinol [107,109,110,111,112,113,114], reducing the clinical state and infectivity to sand flies [115], and in some cases, improving the immune response [116]. Miltefosine is usually employed in dogs with kidney damage (stage IV, according to [91]), as it can decrease proteinuria [117]. As in the treatment of human visceral leishmaniasis, resistance has already been reported in dogs [118], and a study found an increased *L. infantum* resistance to miltefosine and amphotericin B after the treatment of a dog with miltefosine plus allopurinol [118].

Paromomycin, or aminosidine, is an aminoglycosidic antibiotic with antibacterial and antileishmanial activity. Its mechanism of action is unclear as it seems to target mitochondria, but other studies have reported interaction with 30S and 50S ribosomal subunits, blocking protein synthesis [119]. Paromomycin is a second-line treatment that has been positively evaluated in dogs in combination with allopurinol, showing similar or inferior results to the antimonial/allopurinol duo [101,120,121,122].

Amphotericin B is a macrolide widely used in systemic fungal infections. It shows large affinity for ergosterol, the dominant sterol in the *Leishmania* cell membrane, binding it and forming pores that lead to ionic imbalance in the parasite [123]. They are highly effective in human leishmaniases, particularly in their lipid formulations (AmBisome) that have reduced toxicity. Resistance mechanisms have already been reported, in which the parasite produces non-alkylated ergosterol [124]. This compound is not widely used in CanL because of the close kidney monitoring required, the need of intravenous administration, and the fact that its efficacy is not completely clear [125,126,127].

Other drugs include pentamidine isethionate, which presents a higher toxicity [93], and marbofloxacine, which has been used in several clinical trials and seems useful in the treatment of CanL [128,129], particularly in cases with chronic kidney disease [130]. Treatment with ketoconazole and combinations including metronidazole, spiramycine, or enrofloxacin, have not been found effective in CanL treatment [93].

Domperidone can be used in the treatment [131] and prevention of CanL [132,133]. This drug is a D2 dopamine agonist that can improve the animals’ immune response through the increase in prolactin that enhances Th1 response, essential in leishmaniotic dogs. The use of this drug is widespread in European countries [134]. Vitamin D has also been associated with disease progression, and its supplementation has been suggested as a treatment adjuvant, but a study is needed to confirm this hypothesis [135,136].

Treatment monitoring is recommended after the first month of treatment, including a full blood test focusing on kidney damage biomarkers, and then every 3–4 months until full recovery. After recovery, the dog’s state should be checked every 6–12 months in order to detect and prevent relapses. Antibody titre can be used to anticipate relapses, but parasite load quantitation has also proved useful in the monitoring of the disease treatment and evolution [91,137,138,139].

Given the neglected nature of leishmaniasis, progress in drug discovery is slow, and the few drugs that reach clinical stages in humans are unlikely to be commercialised in CanL, even though CanL would be a promising model for antileishmanial drugs. Some experimental drugs have been evaluated in CanL, with most of them used in other diseases. In our group, we have successfully evaluated a natural compound and a promising histone deacetylase inhibitor [80,104]. Artesunate, an antimalarial, was successfully evaluated in combination with meglumine antimoniate [140]. Dietary nucleotides and an active hexose correlated compound were found to be superior to the classical antileishmanial combination of meglumine antimoniate and allopurinol in a recent clinical trial [141]. Immunotherapy has been also successfully tried using anti-IL10 in dogs [142].

## 7. Prevention Measures and Vaccines

Sand flies are the only proven vectors of *Leishmania infantum*, so measures focusing on reducing their density and vectorial function are the best methods to prevent infection [143]. Recent surveys in Mediterranean Europe reported that repellent (deltamethrin, flumethrin, fipronil, or permethrin) use is widespread in these countries, usually combining collars with pipette administration. This repellent use was reported to be more frequent than vaccination, which is viewed as a second-line strategy [144]. Deltamethrin and permethrin have been found useful against *Phlebotomus perniciosus*, but new studies and standardised procedures are needed to establish the susceptibility of leishmaniasis vectors against these agents [145,146]. Deltamethrin was also proved effective in Greece against *P. perfiliewi*, and a long-term advantage was reported when using the ultra-low volume spray method [147]. Long-term overexposure to pyrethroids generates resistance, as found in *P. papatasi* and *P. tobbi* [148]. One of the major drawbacks of the use of insecticides is the toxic effect over the sand flies environment. Natural origin repellents are an alternative for that limitation; however, most of these compounds have been tested in vitro regarding stages with little practical value, or their effect was transient and unsustainable in the long term [149]. The most effective preventive strategy would be the combination of insecticide/repellent and an effective vaccine, even though currently available vaccines can only partially protect against infection [150]. Other strategies are based in the limitation of the host-vector interaction and the reduction of vector feeding microhabitats, particularly those where dogs rest during vector peak activity hours [151].

The development of an effective vaccine against leishmaniasis has been an ambitious goal in the field of neglected tropical diseases for years. Despite the advantages of an effective anti-CanL vaccine, some issues in the field of diagnosis and epidemiology would arise from it: anti-*L. infantum* antibodies induced by the Canileish^®^ vaccine can be detected using IFAT in 3% of dogs up to 1 year after vaccinations, obscuring the diagnosis of naturally infected dogs [150]. Some antibodies can be specific to the vaccine and not detectable by the usual serological tests, whereas others can cause cross-reactions [152]. The generation of residual antibody titres below the serological threshold is another possibility that would lead to unclear diagnosis in asymptomatic dogs, the biggest leishmaniotic dog population [33]. As a consequence, serological tests should be updated in order to differentiate between infected and vaccinated dogs [153]. Grimaldi et al. (2017) found a differential isotype humoral immune response in vaccinated dogs with CanL, which could be used to determine whether a dog is vaccinated or infected [154].

On the other hand, the induction of the cellular immune response would help control the disease progression and in addition, would reduce the parasite load in the dog reservoir, decreasing the transmission to the vector [155]. The ability of Canileish^®^ to reduce the infectivity rate and parasite load in sand flies has already been assessed, leading to a low infection rate of *P. perniciosus* [156].

Th1, or cell immune response induction, usually leads to disease control, whereas the humoral, or Th2, response is associated with disease progression and death [143]. Th1 stakeholders are dendritic cells-primed CD4+ and CD8+ lymphocytes that can trigger IFN-γ, IL-12, and TNF- α production, whereas Th2 is associated with IL-4, IL-5, and IL-13. The protective response induces classical macrophage activation that leads to nitric oxide synthesis and parasite destruction, whereas, under Th2 response, the macrophage is activated through the alternative route, increasing arginase activity and leading to parasite survival [157,158]. These immune responses are not pure, and there is a balance between them, usually leaning towards one side or the other. This mixed response restricts the selection of biomarkers to monitor disease progression and immune response induced by vaccines, thus hampering their efficacy assessment [159,160]. In addition, variability is the norm in CanL, and the dogs’ immune response is complex and multifactorial in infected and vaccinated dogs. This variability has often been attached to the nutritional and immunological state of dogs, dog age and breed, and to the parasite strain [158]. This instability has also been reported in CanL diagnosis.

First-generation vaccines (live-attenuated/inactivated vaccines) were initially envisioned as the most effective option, however, transgenic techniques brought about significant advances through vaccines based on attenuated but immunogenic organisms. Selected targets are usually those genes identified as virulence factors [161]. This first generation induced a variety of responses, ranging from good to no protection through non-lasting immunity [162]. The second generation of antileishmanial vaccines was designed while looking for a more stable and specific response through the use of whole crude antigens, purified fractions of *Leishmania* antigens, or recombinant *Leishmania* antigens [163]. Currently commercialised vaccines belong to this group.

To date, 4 vaccines have been commercialised: Leishmune^®^ (2004, Fort Dodge Wyeth, now Zoetis, Sao Paulo, Brazil), Leish-Tec^®^ (2007, Hertape Calier Saúde Animal, now Ceva, Paulinia, Brazil), CaniLeish^®^ (2011, Virbac, Carros, France), and Letifend^®^ (2016, Laboratorios LETI, S.L.U., Barcelona, Spain). Of these, only two were licensed in Europe, CaniLeish^®^ and LetiFend^®^. Leishmune^®^ was a second-generation vaccine made of *L. donovani* fucose-mannose ligand (FML) that was withdrawn by the Brazilian government in 2014 due to low effectiveness in phase III clinical trials (Brazilian Ministry of Agriculture, Brasília, Brazil, 2014), much poorer than its promising initial evaluation (76–80% efficacy). Leish-Tec^®^ is a vaccine constituted of *L. donovani* amastigote A2 recombinant antigen and is the only authorised vaccine in Brazil. Both vaccines induced a marked increase in IFN-γ levels during the first few months post-vaccination, along with an increase in IgG2 titres. IL10 levels are unaffected, or decrease after the use of FML vaccines [164].

CaniLeish^®^ was the first licensed vaccine in Europe in 2011, aimed at the seronegative dog population. This is a second-generation vaccine constituted of purified *L. infantum* excretion-secretion proteins (LiESP). Recent studies highlighted that the vaccine induced IFN-γ production one month after vaccination, but this response disappears after nine months. In addition, Velez et al. could not find differences between the vaccinated and the control group in a recent clinical study regarding the expression and severity of the disease during the first year post-vaccination [164]. On the other hand, another group reported this vaccine as the best option for the prevention of CanL, even when the efficacy numbers were estimated at 25% [165]. CaniLeish^®^ was evaluated with different adjuvants, leading to effectiveness that varied from 68%, using *Quilaja saponaria* saponin purified fraction, to 92%, using muramyl dipeptide [166,167].

LetiFend^®^ was approved in Europe in 2016, and it used purified recombinant protein, known as protein Q, constituted by five epitopes from four *L. infantum* proteins (LiP2a, LiP2b, LiP0, and histone H2A). This chimeric protein had been previously evaluated for serodiagnosis [33]. Reguera et al. (2016) reported a 72% efficacy [168], but this number was much lower in a recent meta-analysis [165].

Currently, 40% of European dog owners vaccinate their dogs; of these, 61% choose Canileish^®^, while the remaining 39% use Letifend^®^ [144].

Sand fly saliva has been reported as a potent immunomodulator, whose components can facilitate the establishment of *Leishmania* infection at the inoculation site. Therefore, anti-saliva antibodies have been suggested as vaccine adjuvants [162]. Some studies have found anti-saliva antibodies in dogs and other animals and reported them useful to evaluate the effectiveness of vectorial control measures [169]. A combined detection of anti-saliva and anti-*Leishmania* antibodies in endemic regions may help solve problems such as false positive results due to cross-reaction with vaccine-induced antibodies.

Another prophylactic agent commercialised in Europe and widely used is domperidone (Leishguard^®^), discovered as potential antileishmanial treatment; its preventive capacity was also determined [131]. Allopurinol is frequently used as a preventative treatment as well, but due to the side effects of this drug, other alternatives are being investigated, such as nucleotides and active hexose correlated compounds [141].

## 8. Conclusions and Perspective

Canine leishmaniasis is expanding to countries where it was previously unknown due to a number of factors, such as climate change and the import of dogs from endemic areas. Surveillance is necessary in this epidemiological situation in order to determine the extent of the disease in these areas and to monitor the appearance of the vector. This epidemiological effort should be complemented by training health professionals to identify the disease and learn its clinical diagnosis, management, and preventive measures. An appropriate control of imported dogs should help reduce the expansion risk. Several vaccines have been developed in the last decade, and even though their efficacy is limited, these advances will pave the way to the development of better vaccines against leishmaniasis and other disease caused by protozoans. Although new pharmacological tools are available, an ideal scenario would include drugs that can eliminate, or at least drastically reduce, the parasite load in target organs and limit transmission to sand flies, without the side effects of current antileishmanials. This successful chemotherapy will probably be a drug combination that includes immune system adjuvants or supplements in order to limit the appearance of resistance. New developments in canine leishmaniasis will logically be promoted by improvements in the knowledge and treatment of human visceral leishmaniasis.

## Figures and Tables

**Table 1:** Sensitivity and specificity of PCR in common invasive and non-invasive tissues. NA: no available data.

Tissue/Sample	Sensitivity (%)	Specificity (%)	Reference
Hair	99.0, 69.2, 84.6	99.0, 100, NA	[15,73,74]
Skin	74.4	NA	[75]
Conjunctival swab	43.8, 53.8	85.7	[73,76]
Oral swab	15.6	93.3	[72]
Lymph node	43.8, 100.0, 100.0, 77.2	82.3, 100, NA, NA	[15,73,74,76]
Bone marrow	72.7	NA	[15]
Peripheral blood	11.3, 61.5, 30.8, 77.2	96.6, 100, NA, NA	[15,73,74,76]

## Data Availability

Not applicable.
